# A Monkey in Church

**Published:** 1890-12

**Authors:** 


### A MONKEY IN CHURCH.

A pet monkey attended the services at a church in Savannah on a recent Sunday. Jocko looked around in a comical way and jumped upon a window. He sat there for a moment, attracting the attention of every body around. He looked up at the choir and measured the distance between his perch and the gallery, and for an instant seemed to have a mind to go up among the singers. But it was too long a jump and Jocko sprang down on the back of a pew. Then he started up toward the altar, skipping from pew to pew. The ladies hurriedly got out of his way. One young man ducked his head to let the animal go over him, but Jocko lit squarely on his back and startled the worshipper. Jocko was startled, too, but he kept going on until he reached the chancel. A flying leap took him on the altar rail, along which he skipped all the way across the church. The clergyman paused and the sexton ran up with a long pole and poked at the intruder. Jocko started back across the altar rail on a run. From a rail he jumped to a pew back and up into a window and then out, to the great relief of the congregation.

